# Study and validation on mitochondrial and immune-related hub genes in gestational diabetes mellitus based on bioinformatics

**DOI:** 10.3389/fendo.2025.1566249

**Published:** 2025-11-17

**Authors:** Xin Zhao, Yuehan Ma, Jianbin Sun, Xiaomei Zhang

**Affiliations:** Department of Endocrinology, Peking University International Hospital, Beijing, China

**Keywords:** gestational diabetes mellitus, mitochondria, immune, hub gene, bioinformatics

## Abstract

**Background:**

Mitochondria and immune function play pivotal roles in the pathogenesis of gestational diabetes mellitus (GDM). However, the intricate molecular mechanisms underlying their involvement remain elusive. Therefore, this study aimed to elucidate the interaction between mitochondria-related genes (MRGs) and immune-related genes (IRGs) in GDM.

**Methods:**

In this study, GDM-related datasets (GSE103552, GSE154414, and GSE173193) were integrated along with MRGs and IRGs. Differential expression analysis was conducted on GSE103552 to identify differentially expressed genes (DEGs), which were then intersected with MRGs and IRGs. Correlations among the intersection genes were evaluated, and those with statistical significance and strong correlation were selected as candidate genes. Three machine learning algorithms were subsequently applied to further refine the selection of signature genes. The optimal model was determined, and genes within this model were designated as signature genes. Expression levels of these genes were then examined, and those showing significant differences and consistent trends between GDM and control groups in both GSE103552 and GSE154414 datasets were identified as hub genes. Further analyses included chromosomal and subcellular localization, enrichment, regulatory mechanism, and drug prediction analyses of hub genes. Key cell types were analyzed in GSE173193. Finally, the expression of hub genes was validated by reverse transcription quantitative polymerase chain reaction (RT-qPCR).

**Results:**

Comprehensive analysis identified MRPL15, MRPL22, and MRPS18C emerged as pivotal hub genes, each showing significantly lower expression levels in the GDM group. Chromosomal localization revealed MRPS18C on chromosome 4, MRPL22 on chromosome 5, and MRPL15 on chromosome 8. Subcellular distribution analysis indicated that MRPL15 and MRPL22 were predominantly localized in the nucleus, whereas MRPS18C was mainly cytoplasmic. Enrichment analysis showed that spliceosome, proteasome, Parkinson disease, and ribosome pathways were enriched by the hub genes. Regulatory analysis revealed that YY1 regulated MRPS18C and MRPL22, ARID3A regulated MRPS18C and MRPL15, and FOXC1 regulated MRPL22 and MRPL15. Finally, results of RT-qPCR results confirmed that MRPL15, MRPL22, and MRPS18C were significantly downregulated in the GDM group.

**Conclusion:**

Our findings highlight the significance of MRPL15, MRPL22, MRPS18C, monocytes, and villous cytotrophoblast cells in GDM. These insights provide valuable implications for the diagnosis and potential therapeutic interventions targeting of GDM.

## 1 Introduction

Gestational diabetes mellitus (GDM) refers to abnormal glucose metabolism disorders of varying severity during pregnancy, and is one of the most common pregnancy complications. With changes in lifestyle and dietary patterns, the incidence of gestational obesity and GDM—closely related conditions—has been increasing yearly (1, 2), placing a heavy burden on affected patients. GDM is associated with an elevated risk of adverse pregnancy outcomes, including preeclampsia, preterm birth, postpartum depression, instrumental or surgical delivery, and birth trauma (3). Fetuses born to women with GDM are prone to fetal developmental abnormalities, such as macrosomia and have higher rates of congenital malformations, often accompanied by hypoglycemia and jaundice. Moreover, in the long term, children born to women with GDM have an increased risk of obesity and type 2 diabetes later in life (4). Therefore, the early detection and prevention of GDM are particularly important for maternal and infant health, and it is necessary to continuously explore new biomarkers to provide a theoretical basis for its treatment of GDM.

Mitochondria are the primary site of aerobic respiration in cells, providing energy for essential biological functions. They generate adenosine triphosphate (ATP) through oxidative phosphorylation (OXPHOS) and participate in key physiological processes, such as maintaining energy metabolism homeostasis, regulating cell survival and apoptosis, producing reactive oxygen species (ROS), and modulating calcium synthesis and homeostasis (1). Studies have shown that mitochondrial dysfunction reduces cellular energy utilization rate, and then the decrease of metabolic capacity, eventually leading to the excessive production of ROS production, oxidative stress, and metabolic diseases (such as diabetes) (5). The functions of mitochondria vary depending on the cell type in the unit (6). Screening for mitochondrial mutations and deletion polymorphisms in Asian Indian women with GDM revealed a relationship between mitochondrial mutations and GDM, suggesting that abnormal mitochondrial function plays a crucial role in the development of the disease (7).

The maternal immune system must balance key maternal immune mediators such as macrophages, natural killer (NK) cells, and regulatory T cells (Tregs) to prevent pathological conditions or pregnancy interruption (8). Both interleukin-6 (IL-6) and interleukin-8 (IL-8) are immune factors, and studies have shown that they influence the pathological processes of pregnancy-related diseases, including preeclampsia, GDM, and inflammation (9). Furthermore, studies have shown that in patients with type 1 diabetes and type 2 diabetes have shown that immune cells—including neutrophils, eosinophils, monocytes, NK cells, and lymphocytes—are altered, whether they are related to pregnancy is involved or not, indicating that these cells play an important role in disease pathogenesis of this disease (10). Although extensive research has focused on immune cells in tumors, but there are few studies have explored their roles in gestational metabolic diseases. Importantly, immune status is closely related to mitochondrial function. A key feature of mitochondria is their ability to regulate the activation, differentiation, and survival of immune cells. In addition, mitochondria can release mitochondrial DNA and mitochondrial ROS, among others, to modulate immune cell transcription of immune cells (10).

At present, the pathogenesis of GDM remains incompletely understood. The main contributing factors include insulin resistance, adipocytokine imbalance, inflammatory factor release, and genetic predisposition (11), but the involvement of mitochondrial and immune mechanisms is rarely investigated. To further elucidate the roles of mitochondria and immunity in GDM, this study screened the relevant hub genes associated with GDM, and conducted enrichment, regulatory mechanism, and drug prediction analyses to explore the pathways through which these hub genes act. Additionally, we examined cell populations in GDM at the single-cell level to identify cell types with crucial roles in the disease progression. Through this research design, we aim to better understand the relationships among mitochondria-related genes (MRGs), immune-related genes (IRGs), and GDM, thereby providing a scientific basis and guidance for future clinical practice.

## 2 Materials and methods

### 2.1 Data collection

GDM-related datasets—GSE103552 (sequencing platform: GPL6244) and GSE154414 (sequencing platform: GPL20301)—were obtained from the GEO database. The GSE103552 dataset, which contained 11 GDM and 8 control primary feto-placental arterial cell samples, served as the training set. The GSE154414 dataset included 4 GDM and 4 control placental tissue samples and served as the validation set.

The sample size was mainly limited by the sample collection period and strict sample inclusion criteria. However, for an exploratory study, this sample size meets the basic analytical requirements. Additionally, cross-validation between the two datasets provides a certain degree of reliability. Although the sample types of samples differ, both focus on the placenta—the key target organ in GDM—as the core research object. Thus, these datasets cross-validate gene expression changes from two perspectives—specific functional cells” and “whole tissue—thereby enhancing the comprehensiveness of the results.

GSE211617 was sequenced using the GPL24676 platform and contained two GDM placental tissue samples and two control placental tissue samples, serving as the single-cell RNA sequencing (scRNA-seq) dataset.

A total of 1,136 mitochondria-related genes (MRGs) were obtained from the MitoCarta 3.0 database (https://www.broadinstitute.org/), and 2,660 immune-related genes (IRGs) were collected from published literature (12).

### 2.2 Differential expression analysis

Differential expression analysis was performed to identify differentially expressed genes (DEGs) between GDM and control groups using the limma package (version 3.56.2) (13), with thresholds of *adjusted* p < 0.05 and |log^2^ fold change (FC)| > 0.5. Volcano map and heat maps of DEGs were generated using the ggplot2 (version 3.4.4) (14) and circlize package (version 0.4.15) (15) packages, respectively, to visualize DEG distribution.

It should be noted that, in exploratory studies, excessively strict FC thresholds (e.g., |log^2^FC| > 1) may exclude genes with small fold changes but meaningful biological significance. Therefore, DEG screening of differentially expressed genes (DEGs) in this study adopted a dual-criterion approach combining both FC and statistical significance thresholds, which enhanced the stringency and biological relevance of the analysis.

### 2.3 Identification and analysis of candidate genes

Differentially expressed MRGs (DE-MRGs) and differentially expressed IRGs (DE-IRGs) were obtained by intersecting DEGs with MRGs and IRGs, respectively. The correlation between DE-MRGs and DE-IRGs was assessed using Spearman correlation analysis, and candidate genes were selected using thresholds of p < 0.001 and |correlation coefficient| > 0.6.

Gene Ontology (GO) and Kyoto Encyclopedia of Genes and Genomes (KEGG) enrichment analyses were then conducted to explore the biological functions and pathways of the candidate genes using the clusterProfiler package (version 4.8.2) (16) with the org.Hs.eg.db background gene set in org.Hs.eg.db package (version 3.17.0) (17) (*adjusted* p < 0.05).

To further investigate the protein-level interactions of candidate genes, the STRING database was used to construct a protein–protein interaction (PPI) network (species: Homo sapiens, interaction score ≥ 0.4). The PPI network was visualized using Cytoscape software (version 3.7.1) (18). Four algorithms in CytoHubba were applied to select potential signature genes, and the intersection of the top 30 genes from all four algorithms was identified as the set of candidate signature genes.

### 2.4 Identification of hub genes

To obtain hub genes, three machine learning models—random forest (RF), support vector machine (SVM), and generalized linear model (GLM)—were constructed using the caret package (version 6.0.49) (19). These models were analyzed with the explain function in the DALEX package (version 2.4.3) (20), and the best-performing model was selected. Genes within the optimal model were identified as signature genes.

The expression of signature genes was compared between GDM and control groups was compared using the Wilcoxon test (p < 0.05), and differences were visualized with the ggpubr package (version 0.6.0) (21). Genes showing statistically significant difference and consistent expression trends were identified as hub genes (p < 0.05).

To assess the diagnostic ability of hub genes to distinguish between GDM and control samples, receiver operating characteristic (ROC) curves were plotted for the hub genes was drafted in the GSE103552 and GSE154414 datasets using the pROC package (version 1.18.4) (22).

### 2.5 Localization and function analysis of hub genes

Chromosomal localization of the hub genes was visualized using the RCircos package (version 1.2.2) (23). The FASTA DNA sequences of the hub genes were obtained from the NCBI database. Subsequently, subcellular localization of the hub genes was analyzed using the mRNALocater database.

To explore the potential relationships between hub genes and other genes, a co-expression network of hub genes was constructed using GeneMANIA (http://www.genemania.org/). Functional similarity among hub genes was evaluated by calculating the average semantic similarity between their Gene Ontology (GO) terms with the GOSemSim package (version 2.26.1) (24).

Gene set enrichment analysis (GSEA) was performed to investigate the biological pathways associated with hub genes involved in GDM. In the GSE103552 dataset, correlation coefficients between the expression levels of hub genes and all genes were calculated and ranked. Based on the background gene set, the top five pathways with the smallest adjusted p values were visualized using the clusterProfiler package (adjusted p < 0.05).

PhosphoSitePlus is a comprehensive protein phosphorylation database that contains extensive experimentally validated data, including information on multiple post-translational modifications (PTMs), including phosphorylation, acetylation, and ubiquitination. The hub genes were imported the hub genes into this database to predict potential types of protein post-translational modifications.

### 2.6 Regulatory mechanism analysis and drug prediction

To explore the molecular regulatory mechanisms of hub genes in GDM, transcription factors (TFs) targeting the hub genes were predicted using JASPAR in NetworkAnalyst (https://www.networkanalyst.ca/). In addition, microRNAs (miRNAs) targeting the hub genes were predicted using the ENCORI database (https://rnasysu.com/encori/). Long noncoding RNAs (lncRNAs) targeting the hub genes were obtained from both miRNet (https://www.mirnet.ca/miRNet/home.xhtml) and the ENCORI database. The intersecting lncRNAs from the two databases were identified as key lncRNAs.

Based on the identified hub genes, miRNAs, and key lncRNAs, an lncRNA–miRNA–hub gene regulatory network was constructed and visualized using Cytoscape software.

Furthermore, potential therapeutic drugs for GDM were predicted using the Comparative Toxicogenomics Database (CTD) (https://ctdbase.org/) based on the hub genes. The results were also visualized using Cytoscape software.

### 2.7 scRNA-seq data analysis

The Seurat package (version 5.1.0) (25) was used for scRNA-seq data analysis in the GSE173193 dataset. Cells with fewer than 200 or more than 6,000 genes, genes expressed in fewer than three cells or with counts greater than 50,000, and cells with more than 15% proportion of genes expressed in mitochondria were removed from subsequent analyses. After quality control, the data were normalized using the *NormalizeData* function in the “Seurat package (version 5.1.0). Subsequently, the top 2,000 genes with the highest variability were identified using the FindVariableFeatures function. Next, the dimensionality reduction was performed through principal component analysis (PCA). The ElbowPlot function in the “Seurat” package (version 5.1.0) was used to draw the elbow plot, and the principal components (PCs) before the inflection point were selected for subsequent analysis. Subsequently, Based on the selected PCs, unsupervised clustering (resolution = 0.2) was conducted via uniform manifold approximation and projection (UMAP) for all cells using the FindNeighbors and FindClusters functions of the Seurat” package (version 5.1.0). Annotated analysis of cell clusters was performed to identify specific cell types based on marker genes (26) obtained from the literature. At the same time, the percentage of various cell types was also shown (p < 0.05). Cell types with a significant differences in proportion between GDM placental tissue samples and normal placental tissue samples were identified. Subsequently, key cells were determined based on the differential expression of hub genes in these distinct cell types. Cell–cell communication analysis among cell types was performed using the CellChat” package (version 1.6.1) (27) to study intercellular correlations. Functional enrichment analysis of cell types was carried out using the “ReactomeGSA” package (version 1.16.1) (28). Differentially enriched pathways among different cell types were identified, and the top 10 pathways with the greatest differences were visualized. The Monocle package (version 2.28.0; PMID: 28114287) was used to perform pseudotime analysis of key cells to investigate their differentiation trajectories and the expression changes of hub genes during this transition process of key cells.

### 2.8 Expression analysis of hub genes

A total of five pairs of samples (five control (1–5) and five GDM (6–10) placental samples) from mice were obtained from Peking University International Hospital. The study was approved by the Peking University Health Science Center Animal Ethics Committee (Ethics approval number: PUIRB-LA2023181).

Total RNA from the 10 samples (50 mg each) was extracted using 1 mL TRIzol reagent (Ambion, USA) according to the manufacturer’s protocol. Then the RNA concentration was measured using a NanoPhotometer N50. Complementary DNA (cDNA) was synthesized by reverse transcription using the SureScript First-Strand cDNA Synthesis Kit, and the reverse transcription was performed with an S1000TM Thermal Cycler (Bio-Rad, USA).

Reverse transcription quantitative polymerase chain reaction (RT-qPCR) assay was performed using the CFX Connect Real-Time Quantitative Fluorescence PCR Instrument (Bio-Rad, USA) under the following conditions: pre-denaturation at 95 °C for 1 min; denaturation at 95 °C for 20 s, annealing at 55 °C for 20 s, and extension at 72 °C for 30 s, for a total of 40 cycles. The relative quantification of mRNA levels was calculated using the 2−^ΔΔCT^ method.

### 2.9 Statistical analysis

R software (version 4.2.2) was used for data processing and analysis. Statistical significance between two groups was determined using the Wilcoxon rank-sum test. A *p*-value < 0.05 was considered statistically significant.

## 3 Results

### 3.1 A total of 148 GDM-related candidate genes were screened out

**Figure 1:**
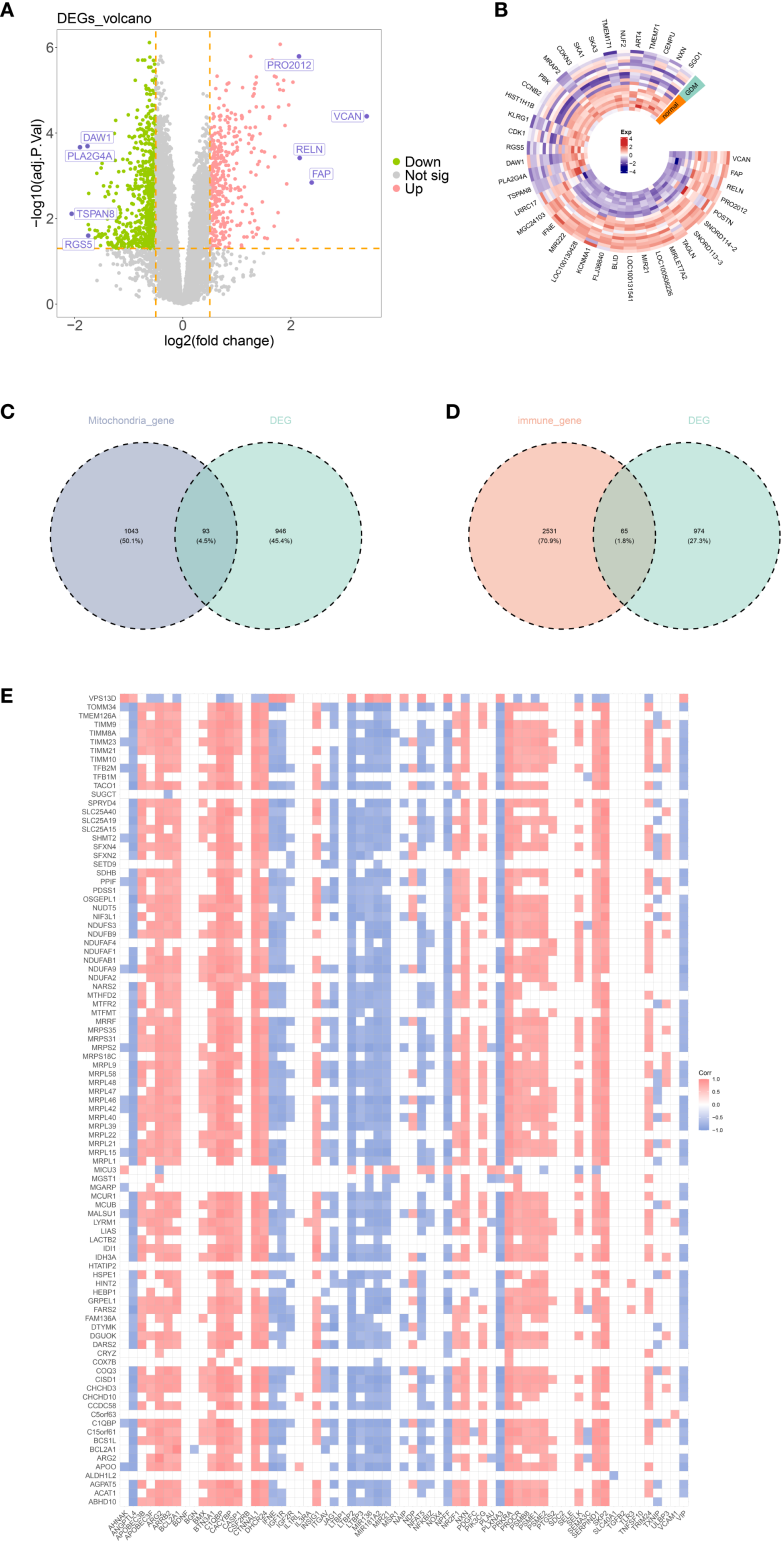
**(A, B)** Differential gene volcano plot and heatmap. **(C, D)** Venn diagram of differentially expressed genes, mitochondrial genes, and immune genes. **(E)** Correlation heatmap between differential mitochondrial genes and differential immune genes. Thresholds for differential analysis were adjusted p-value < 0.05 and |log_2_FC| > 0.5; thresholds for correlation analysis were p-value < 0.05 and |cor| > 0.3.

A total of 1,039 DEGs were identified between the GDM and control groups in the GSE103552 dataset. Among these, 391 genes were upregulated and 648 genes were downregulated (**Figures 1A, B**). By overlapping the 1,039 DEGs with 1,136 MRGs and 2,660 IRGs, 93 DE-MRGs and 65 DE-IRGs were obtained, respectively (**Figures 1C, D**). After calculating the correlations between the 93 DE-MRGs and 65 DE-IRGs, 148 candidate genes were finally screened out (p < 0.001 and |cor| > 0.6) (**Figure 1E**).

### 3.2 Screening of candidate signature genes in GDM

**Figure 2:**
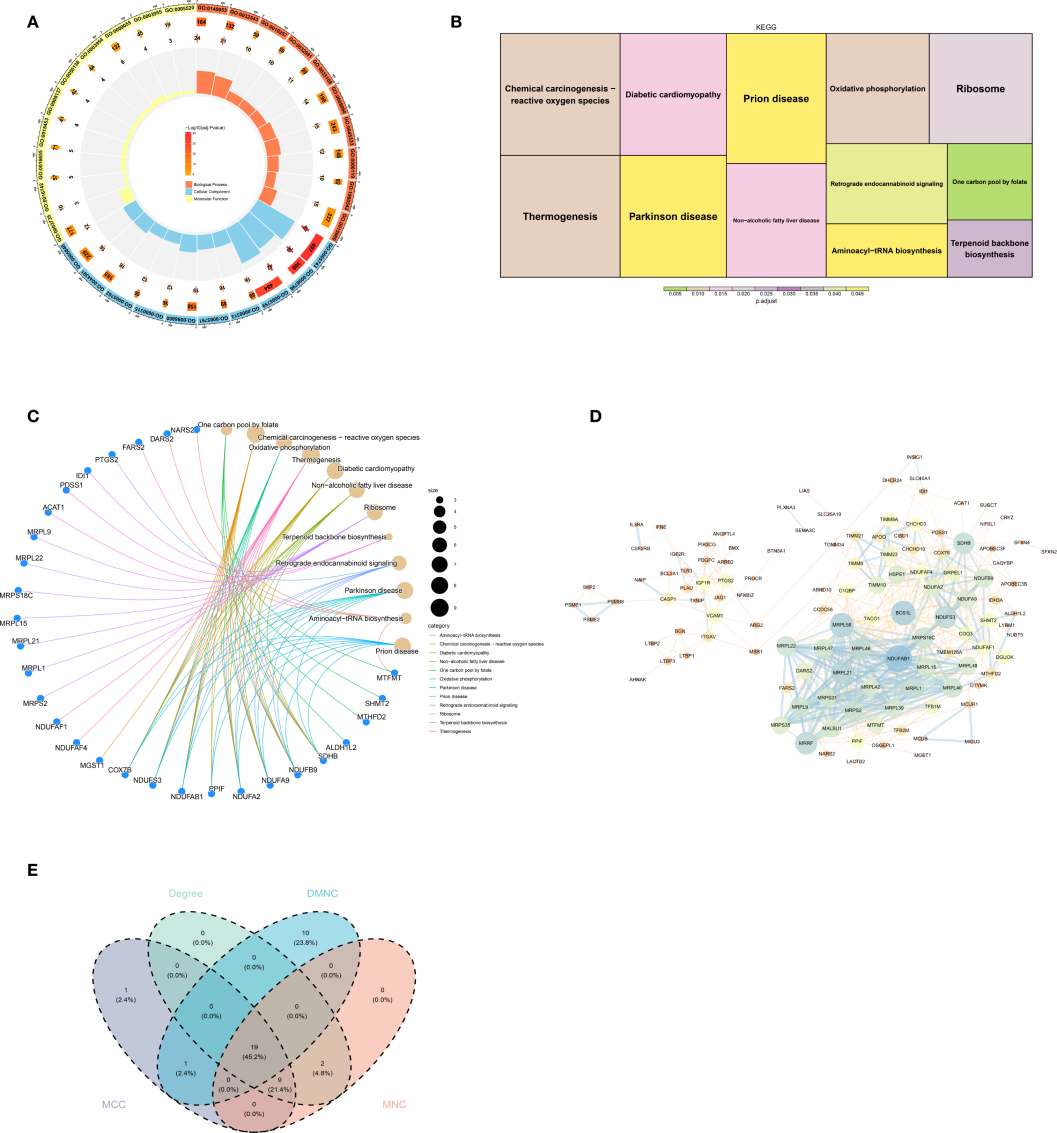
**(A)** GO enrichment circle diagram. **(B, C)** KEGG enrichment diagrams. **(D)** PPI network diagram. **(E)** CytoHubba screening gene Venn diagram.

To identify the biological functions and pathways associated with the candidate genes, GO and KEGG enrichment analyses were performed. The results showed that 132 GO terms were significantly enriched, including mitochondrial gene expression, mitochondrial inner membrane, and structural constituent of the ribosome, etc. were enriched (**Figure 2A**). Furthermore, the candidate genes were enriched in 12 KEGG pathways, involving in chemical carcinogenesis–reactive oxygen species, thermogenesis, and related processes (**Figures 2B, C**).

A PPI network was constructed containing 119 nodes and 535 edges. NDUFAB1 exhibited the highest degree of connectivity with other genes (**Figure 2D**). By intersecting the top 30 genes from four algorithms, 19 candidate signature genes—including MRPS18C, MRPL22, and MRPL15—were obtained (**Figures 1A–D**, **Figure 2E**).

### 3.3 MRPL15, MRPL22 and MRPS18C were identified as hub genes

**Figure 3:**
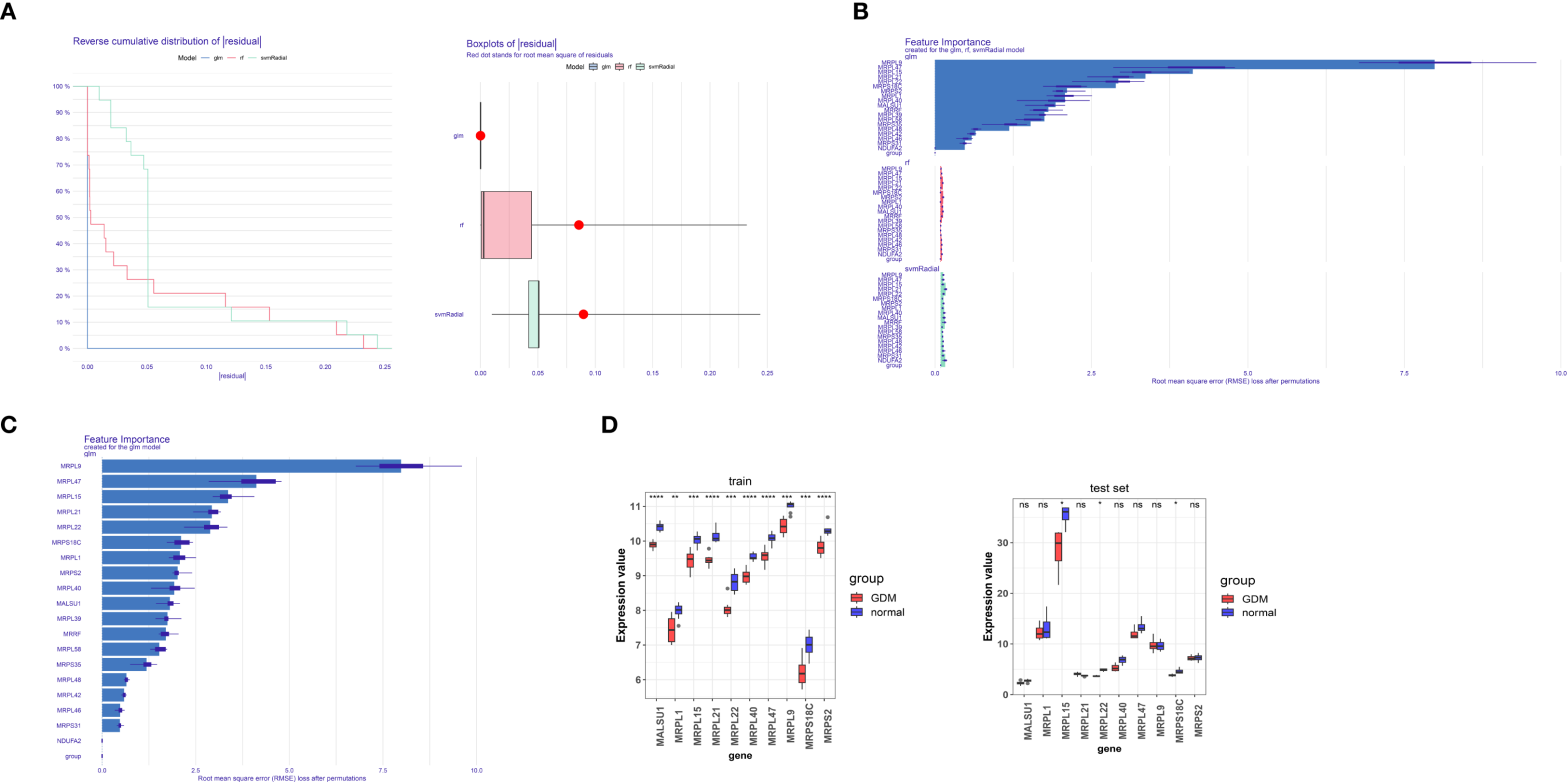
**(A)** Evaluation of machine learning models by sample cumulative residual distribution plots and sample residual box plots. **(B, C)** Importance of explanatory variables in the three models **(B)** and in the best model **(C)**. **(D)** Box plots of candidate gene expression levels. * represent p < 0.05, ** represent p < 0.01, *** represent p < 0.001, **** represent p < 0.0001.

After analyzing the RF, SVM, and GLM models, the GLM model was determined to be the best-performing model (**Figures 3A, B**). The top 10 genes in this model (MRPL9, MRPL47, MRPL15, MRPL21, MRPL22, MRPS18C, MRPL1, MRPS2, MRPL40, and MALSU1) were identified as signature genes (**Figure 3C**).

Expression analysis revealed that MRPL15, MRPL22, and MRPS18C had higher expression levels in the control group than in the GDM group in both the GSE103552 and GSE154414 datasets. Therefore, MRPL15, MRPL22, and MRPS18C were identified as hub genes (**Figure 3D**).

### 3.4 Corresponding localization and pathways of hub genes in GDM

**Figure 4:**
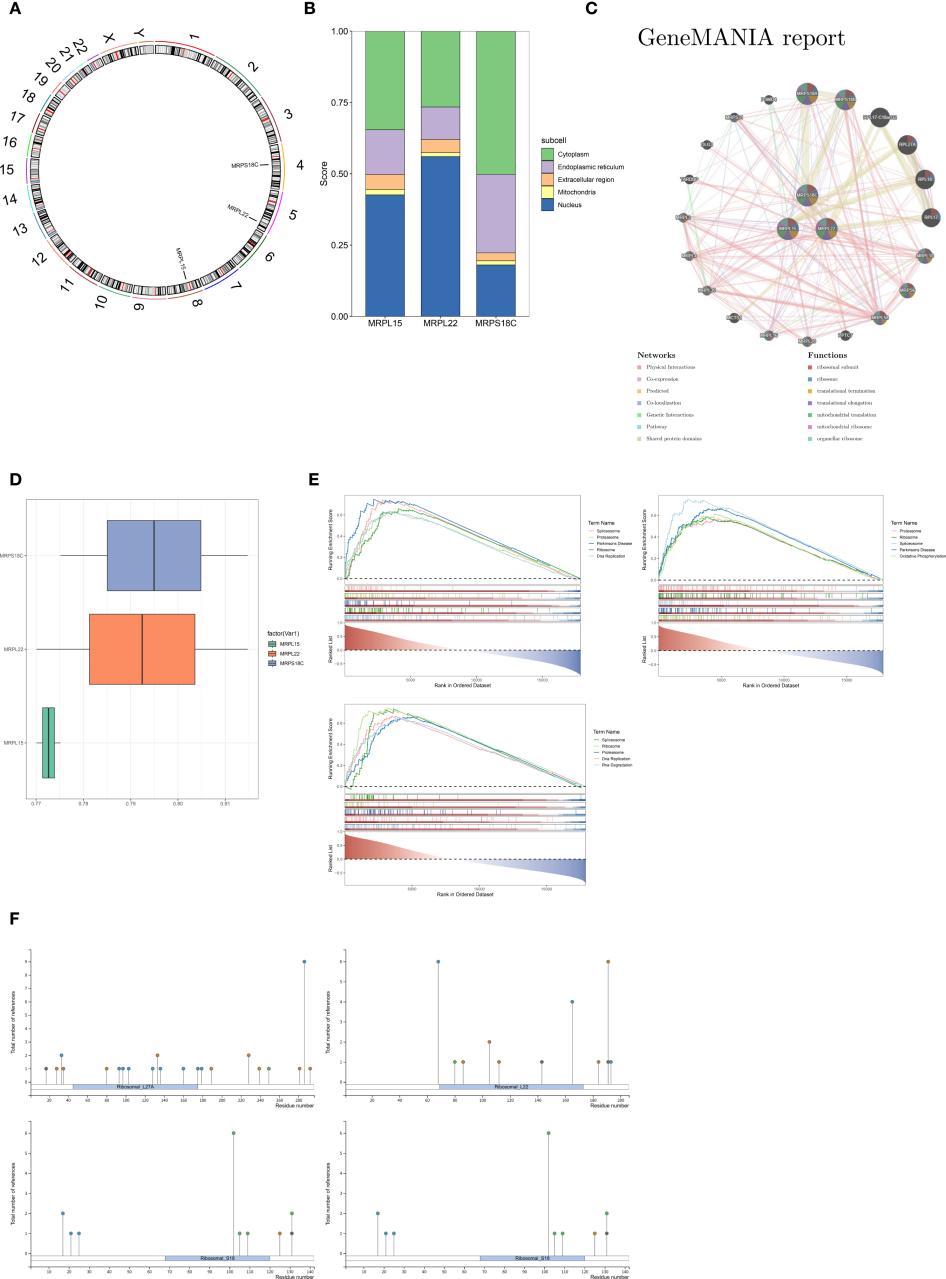
**(A)** Chromosomal localization of hub genes. **(B)** Subcellular localization of hub genes. **(C)** GeneMANIA network. **(D)** Functional similarity analysis of hub genes. **(E)** GSEA results. **(F)** Post-translational modification (PTM) analysis of hub genes.

Chromosomal localization analysis showed that MRPS18C was located on chromosome 4, MRPL22 was on chromosome 5, and MRPL15 was on chromosome 8 (**Figure 4A**). Subcellular localization analysis indicated that MRPL15 and MRPL22 were mainly expressed in the nucleus (proportion > 40%), whereas MRPS18C was mainly expressed in the cytoplasm (proportion > 50%) (**Figure 4B**).

The hub genes were found to share similar functions with MRPS18A, MRPS18A, RPL17-C18orf32, and other ribosomal proteins. Their main functions included the ribosomal subunit, ribosome, and translational termination processes (**Figure 4C**). Similarity analysis showed that MRPS18C and MRPL22 had higher functional similarity than MRPL15 (**Figure 4D**).

Additionally, GSEA was performed to explore biological pathways involving the hub genes in GDM. The top five pathways were enriched in spliceosome, proteasome, and ribosome-related processes (**Figure 4E**). Based on the PhosphoSitePlus database, we predicted the post-translational modification (PTM) types of the hub genes were predicted: MRPL15 was mainly modified by phosphorylation and ubiquitination, MRPL22 was primarily subject to phosphorylation and ubiquitination, and MRPS18C was mainly modified by phosphorylation and acetylation (**Figure 4F**).

### 3.5 Gene regulatory networks and potential drugs of hub genes in GDM

**Figure 5:**
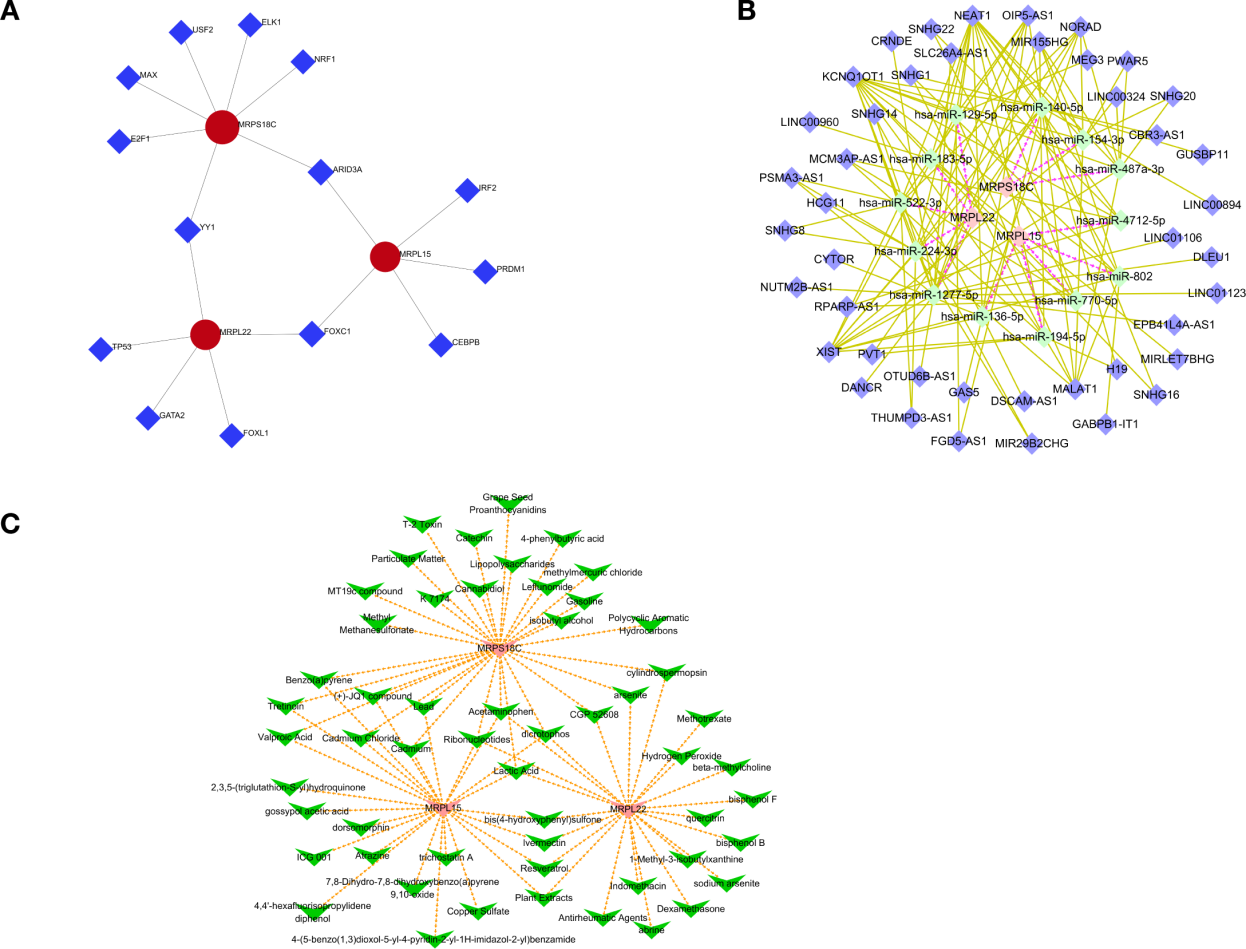
**(A)** Transcription factor (TF) network diagram. **(B)** mRNA–miRNA–lncRNA relationship network diagram. **(C)** Network diagram showing the relationship between genes and drugs.

To clarify the regulatory mechanisms of hub genes in GDM, 11 transcription factors (TFs) were predicted. Among these TFs, YY1 regulated MRPS18C and MRPL22; ARID3A regulated MRPS18C and MRPL15; and FOXC1 regulated MRPL22 and MRPL15 (**Figure 5A**). According to the hub genes, 13 miRNAs and 43 lncRNAs were obtained, and an lncRNA–miRNA–hub gene network was constructed with 59 nodes and 128 edges. OIP5-AS1, NEAT1, and KCNQ1OT1 regulated MRPL22 through hsa-miR-1277-5p, hsa-miR-129-5p, hsa-miR-183-5p, hsa-miR-224-3p, and hsa-miR-522-3p. NEAT1, MALAT1, KCNQ1OT1, and XIST regulated MRPS18C through hsa-miR-140-5p, hsa-miR-154-3p, and hsa-miR-487a-3p. NEAT1 and KCNQ1OT1 regulated MRPL15 through hsa-miR-136-5p, hsa-miR-194-5p, hsa-miR-4712-5p, hsa-miR-770-5p, and hsa-miR-802 (**Figure 5B**).

Furthermore, potential drugs for GDM were predicted based on the hub genes. Acetaminophen, dicrotophos, lactic acid, and ribonucleotides were simultaneously predicted to target MRPL15, MRPL22, and MRPS18C (**Figure 5C**).

### 3.6 Cells were clustered into nine types

**Figure 6:**
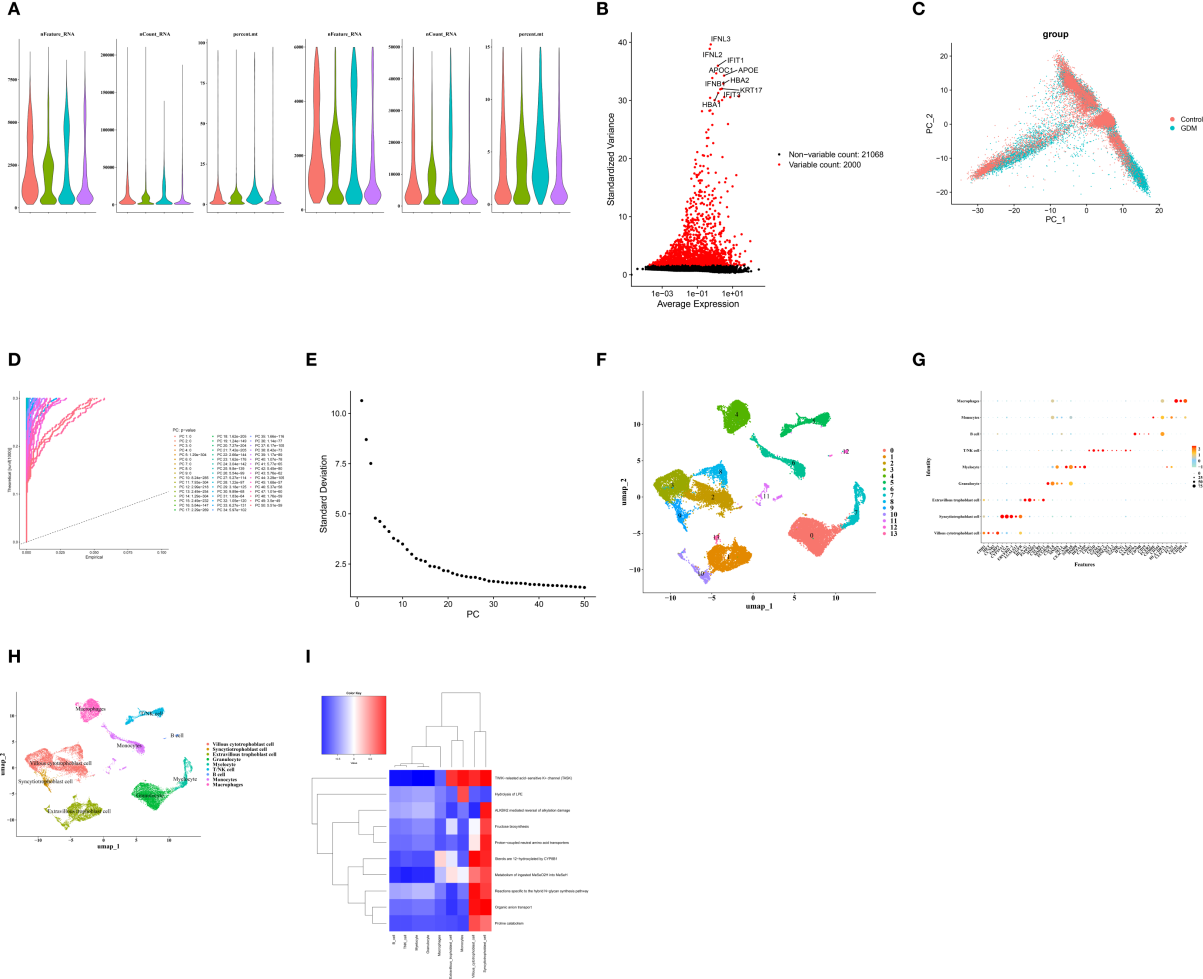
**(A)** Violin plots of the single-cell dataset before and after quality control. **(B)** Screening of highly variable genes. **(C–E)** Principal component analysis (PCA). **(F)** Cell clustering results. **(G, H)** Dot plot of marker gene expression in each cell type **(G)** and cell annotation results **(H)**. **(I)** Enrichment analysis of each cell type.

To explore the cell populations associated with GDM, scRNA-seq analysis was performed. After quality control, a total of 25,487 cells and 23,068 genes were retained (**Figure 6A****),** and the top 2,000 highly variable genes were identified (**Figure 6B**). In PCA, 30 principal components (PCs) were selected for subsequent analyses according to the elbow plot (**Figures 6C–E**). The cells were then clustered into 14 clusters (**Figure 6F**).

Based on marker gene expression of marker genes, the clustered cells were classified into nine cell types: villous cytotrophoblast cells, syncytiotrophoblast cells, extravillous trophoblast cells, myelocytes, T/NK cells, B cells, monocytes, macrophages, and granulocytes (**Figures 6G, H**). Functional enrichment analysis of the nine cell types of cells was conducted to identify the pathways in which they were involved. The top 10 pathways showing the largest differences were visualized, including the TWIK-related acid-sensitive K^+^ channel, hydrolysis of LPE, and ALKBH2-mediated reversal of alkylation damage, etc (**Figure 6I**).

### 3.7 Monocytes and villous cytotrophoblast cells were further defined as key cells

**Figure 7:**
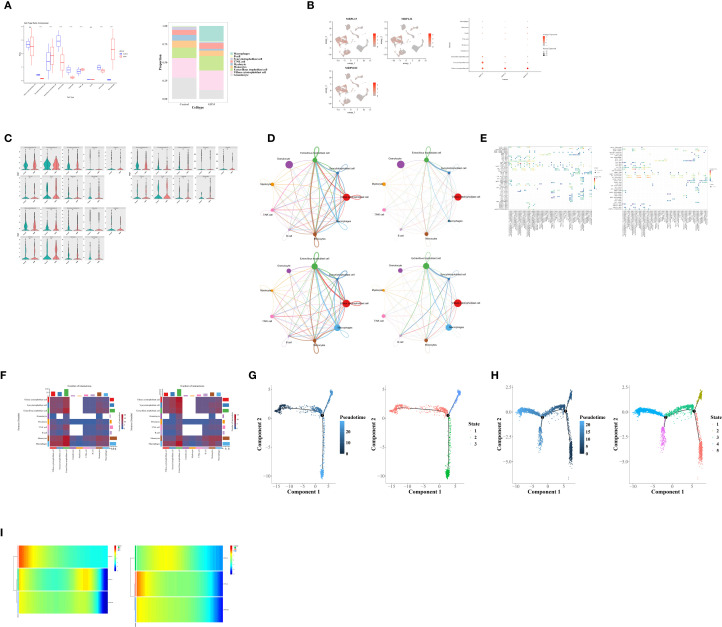
**(A)** Identification of differential cells between the GDM and control groups. **(B)** Expression of hub genes in each cell type. **(C)** Differential expression analysis of hub genes in each cell type (for identification of key cells). **(D)** Number and intensity of cell–cell communications between the control and GDM groups (1–2 represent communication number and intensity of the control group, respectively; 3–4 represent communication number and intensity of the GDM group, respectively). **(E)** Dot plot of cell–cell communication receptor–ligand pairs between the control and GDM groups. **(F)** Heatmaps of intercellular interaction networks in the control and GDM groups. **(G, H)** Pseudotime differentiation trajectories of key cells: monocytes **(G)** and villous cytotrophoblasts **(H)**. **(I)** Expression trends of hub genes during differentiation of key cells: monocytes (1) and villous cytotrophoblasts (2).

We first identified seven differential cell types between the GDM group and the control groups (**Figures 7A, B**). MRPL15 showed a significant expression difference in monocytes; MRPL22 exhibited a significantly higher expression difference in villous cytotrophoblast cells; and *MRPS18C* displayed significant expression differences in villous cytotrophoblast cells, monocytes, and granulocytes (**Figure 7C**). Therefore, monocytes and villous cytotrophoblast cells were selected and defined as key cells.

Next, the intercellular interaction network among all cells in the GDM and the control groups was analyzed. The results showed that, compared with the control group, the number of interactions between monocytes, T/NK cells, and other cells decreased in the GDM group decreased (**Figure 7D**). In addition, the receptor–ligand pairs MIF–(CD74+CXCR4) and MIF–(CD74+CD44) were more active in the GDM group than in the control group (**Figure 7E**). A heatmap of the intercellular interaction network further indicated that the total number of intercellular interactions was reduced in the disease group was reduced compared with the control group (**Figure 7F**).

Pseudotime analysis was then conducted for the key cells. During the differentiation and development of monocytes, one developmental node and three differentiation states were identified (**Figure 7G**). For villous cytotrophoblast cells, two developmental nodes and five differentiation states were observed during their differentiation and development (**Figure 7H**). The expression levels of MRPL15, MRPL22, and *MRPS18C* all showed a decreasing trend during the differentiation of both monocytes and villous cytotrophoblast cells (**Figure 7I**).

### 3.8 Expression analysis results

**Figure 8:**
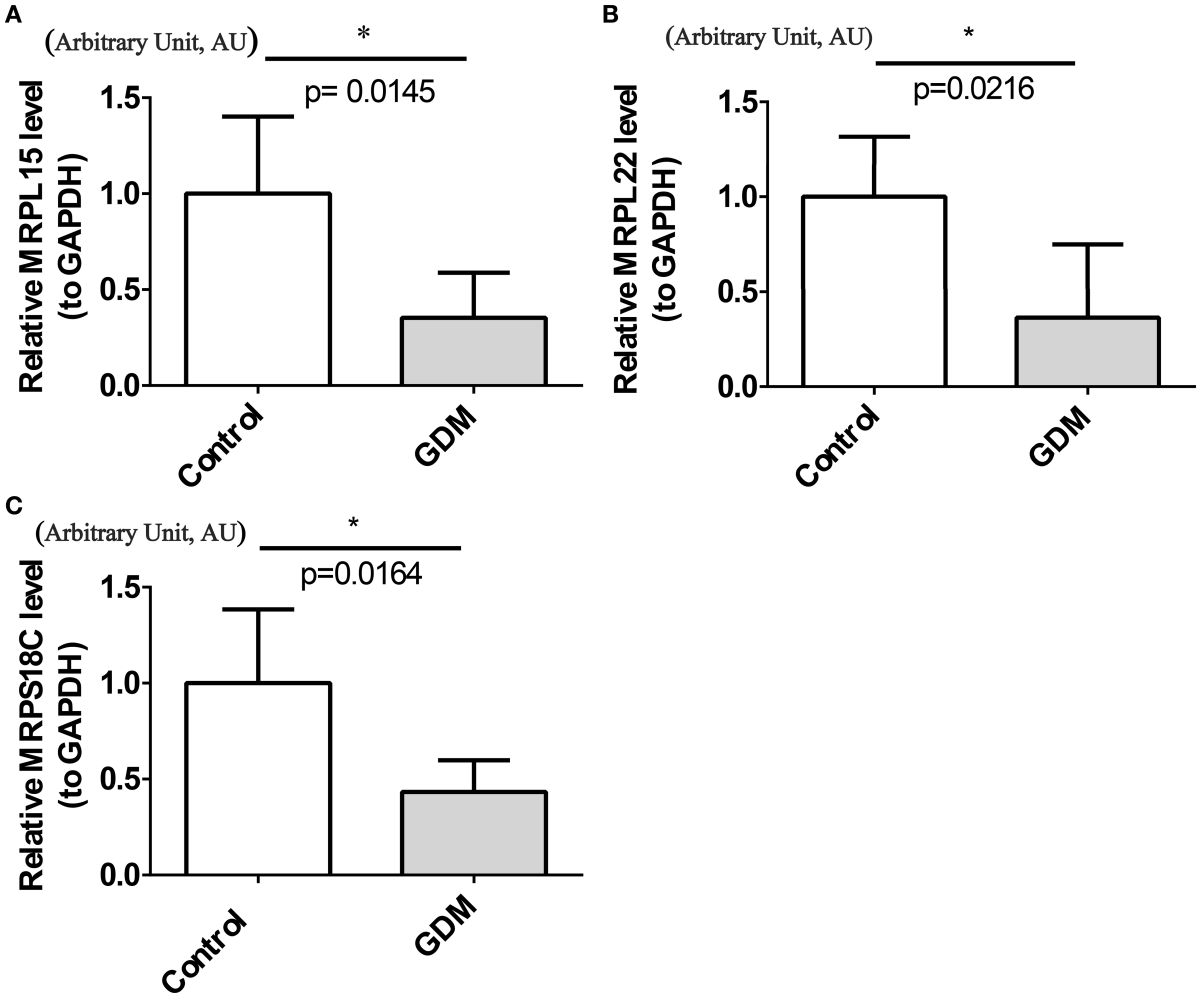
Confirmation of hub DEG expression in GDM mice. **(A)** MRPL15 expression in control (CON) and GDM mice. **(B)** MRPL22 expression in CON and GDM mice. **(C)** MRPS18C expression in CON and GDM mice. p < 0.05.

RT-qPCR results showed that MRPL15, MRPL22, and MRPS18C had significantly lower expression levels in the GDM group (**Figures 8A–C**).

## 4 Discussion

With changes in social and economic life and dietary structure, the incidence of gestational obesity and its closely related GDM is increasing year by year (29). Studies have shown that the expression levels of mitochondrial electron transfer complexes I, II, III, and IV in GDM women with GDM treated with insulin or oral hypoglycemic drugs are lower than those in GDM women treated with normal pregnancy or those treated with diet control (30). Other studies have shown that some immune cells, including neutrophils, eosinophils, monocytes, NK cells, and lymphocytes, are regulated in patients with type 1 diabetes mellitus and type 2 diabetes mellitus, whether related to pregnancy-related or not, indicating that these cells play an important role in the pathogenesis of this disease (10). In this study, the hub genes related to mitochondria and immunity in the process of GDM, as well as the biological processes and mechanisms involved, were analyzed by bioinformatics to provide a theoretical basis for the treatment of GDM.

In this study, 1,093 differentially expressed genes between the GDM group and the normal group were screened, including 391 upregulated genes, 698 downregulated genes, and 148 candidate genes. Subsequently, GO and KEGG functional enrichment analyses were performed to obtain pathways such as mitochondrial gene expression, mitochondrial translation, NADH dehydrogenase complex assembly, and carbon pool by folate, chemical carcinogenesis–reactive oxygen species, and oxidative phosphorylation and so on. Previous studies have shown that the correlation between GDM and PM2.5 may be attributed to the possibility that high PM2.5 levels inducing mitochondrial gene dysfunction. Mitochondrial OXPHOS dysfunction affects the active growth of related genes and leads to mitochondrial damage in healthy premature infants (including newborns) through the changes in electron transport chain complex proteins (31). Another study found that endothelial dysfunction may be one of the mechanisms of GDM by comparing the difference of superoxide differences between GDM and healthy umbilical vein endothelial cells (32).

In this study, three hub genes in GDM were identified by machine learning and expression validation: MRPL15, MRPL22, and MRPS18C. MRPL15 belongs to the mitochondrial biomarker set of genes, which may encode mammalian mitochondrial ribosomal proteins and thus assist in protein synthesis within the mitochondrion. Previous research has shown that MRPL15 can be used as a companion diagnostic marker to determine which breast cancer patients might benefit most from clinical therapy (33). As a risk gene and potential biological target of Alzheimer’s disease (AD), MRPL15 also plays an important role in regulating immune cells in AD (34). In addition, a recent study has confirmed that MRPL15 is significantly correlated with diabetic retinopathy (35). However, the abnormal expression of MRPL15 in GDM has not been confirmed. Previous studies have shown that MRPL22, as an immune-related gene, participates in the T cell receptor signaling pathway and was identified as a hub gene for the diagnosis of ischemic stroke (36). Recent studies have also shown that MRPL22 was identified as a shared gene signature for endometrial cancer and polycystic ovary syndrome (37). The MRPS18C gene belongs to the mitochondrial ribosomal protein (MRP) family, which is involved in mitochondrial translational termination, elongation, translation, and poly (A) RNA binding. Studies have shown that MRPS18C is negatively correlated with overall survival in breast cancer and may act as a biomarker for risk prediction and may serve as a potential genetic target in breast cancer patients (38). However, there is no known correlation between these three genes and the occurrence of GDM either domestically or internationally. This study is the first time to find that differences in the difference of expression of these three genes may contribute to the occurrence of GDM.

In this study, GSEA enrichment was used to explore the pathway functions of the hub genes. The results showed that the hub genes were mainly concentrated in the proteasome and Parkinson’s disease (PD) pathways. Misfolded proteins are usually degraded by the ubiquitin–proteasome system. However, if this system is damaged, misfolded proteins will escape degradation and are released into the cytoplasm. Maternal hyperglycemia can lead to abnormal gene expression in the proteasome, resulting in the accumulation of misfolded cytotoxic proteins in cells and impaired organelle function. This may induce mitochondria to produce a large amounts of ROS, leading to oxidative stress and intracellular signaling disturbances that alter cell activity (39). Furthermore, studies have shown that gestational factors play an important role in shaping brain development. GDM may cause interindividual variation in neuronal and glial cell load at birth, potentially influencing acquired neurodegenerative diseases, including PD and Alzheimer’s disease (AD) (40).

One of the core pathological features of Parkinson’s disease (PD) is mitochondrial dysfunction in substantia nigra dopaminergic neurons, which is specifically manifested by decreased activity of mitochondrial respiratory chain complex I (NADH dehydrogenase), mitochondrial DNA (mtDNA) damage, excessive accumulation of reactive oxygen species (ROS), and ultimately neuronal apoptosis (41, 42). Similar mitochondrial pathological phenotypes have been reported in GDM placentas (43, 44). Hub genes such as *MRPL15* and *MRPL22* are enriched in the PD pathway, linking PD and GDM. We speculate that they share a core pathological mechanism of “mitochondrial functional defect–oxidative stress imbalance.” MRPL15 and MRPL22 are both mitochondrial function–related genes. As core subunits of the mitochondrial ribosome, they are essential for mitochondrial oxidative phosphorylation and play crucial roles in regulating cell death–inducing factors (45–47). Abnormal expression of mitochondrial ribosomal proteins (MRPs) can lead to various disorders, such as mitochondrial metabolic defects and cellular dysfunction. Changes in the expression of these genes directly trigger a chain reaction of “decreased mitochondrial translation efficiency → OXPHOS complex assembly defect → decreased mitochondrial respiratory function → ROS accumulation,” which represents not only the core pathogenesis of PD but also the key molecular basis of placental dysfunction in GDM.

In this study, monocytes and villous cytotrophoblast cells were identified as key cells in GDM. Monocytes are important innate immune cells in the maternal circulation. They can contribute to the pathological process of GDM by differentiating into macrophages (such as extravillous macrophages in placental tissue), secreting inflammatory factors, and regulating metabolism-related pathways. Studies have shown that the monocyte-to-lymphocyte ratio in early pregnancy is a predictor of GDM (48). These activated monocytes oversecrete proinflammatory cytokines (such as IL-6, TNF-α, and IL-1β) (49), and there is a close link between the production of inflammatory biomarkers and the occurrence of GDM (50).

Villous cytotrophoblasts (VCTs) are the core cell type of placental villous lobules. Their main functions include differentiation into syncytiotrophoblasts (STBs), transport of materials (glucose, amino acids, and fatty acids), secretion of placental hormones (such as hCG and placental lactogen), and participation in placental vascularization (51, 52). Studies have shown that lipopolysaccharide (LPS) with 2.5–25 mM glucose can induce increased expression of autophagy proteins, inflammatory markers, and m6A levels in human villous trophoblasts (53). GDM alters the balance of paracrine factors regulating trophoblast-derived angiogenesis, which may lead to GDM-related pathological changes in placental angiogenesis and vascular structure (54). Under normal circumstances, placental development requires proper coordination of trophoblast proliferation, differentiation, and invasion, whereas in the context of diabetes, trophoblast proliferation, cell death, and cell-cycle control are altered (55). Both previous studies and our findings indicate the important role of these two cell types in the pathogenesis of GDM.

The regulatory network is a key component of the gene expression regulation process. It has important research value, and can reveal the complexity and diversity of gene expression regulation, thereby enabling a deeper understanding of the regulatory mechanisms involved. To study the potential regulatory mechanisms of the final hub genes in GDM, this study further constructed a regulatory network of these hub genes. Eleven TFs were predicted. The TF shared by MRPS18C and MRPL22 was YY1; the TF shared by MRPS18C and MRPL15 was ARID3A; and MRPL22 and *MRPL15* shared FOXC1. The GL-3/FOXC1 pathway has been shown to protect HTR-8/SVneo cells from high glucose–induced apoptosis (56), suggesting that GL-3 and FOXC1 may play important protective roles in hyperglycemia during pregnancy. Studies have also shown that inactivation of YY1 impairs mitochondrial OXPHOS activity in mouse models and induces mitochondrial dysfunction and diabetes (57).

According to the hub genes, 13 miRNAs and 43 lncRNAs were identified. A recent study reported that the level of OIP5-AS1 levels decreased in GDM women with GDM. The OIP5-AS1/miR-137-3p/EZH2 axis may function in HTR-8/SVneo cells under high-glucose conditions (58), suggesting that OIP5-AS1 could be a potential target for the prevention and treatment of GDM. CEBPB is an important transcription factor involved in regulating immune inflammation and metabolic responses, playing significant roles in lipogenesis, glucose and lipid metabolism, liver regeneration, and hematopoiesis. Results have shown that the AKT phosphorylation level of insulin and glucose uptake in hepatocytes were significantly increase when CEBPB expression is eliminated by LIN (59). In addition, recent studies have confirmed that inhibiting the expression of CEBPB in trophoblasts can significantly enhance the insulin signaling by increasing AKT phosphorylation levels in the insulin pathway (60). These findings suggest that CEBPB affects glucose uptake by inhibiting AKT phosphorylation, which may further contribute to the development of GDM.

The miR-194-5p is a multifunctional miRNA involved in regulating cell differentiation and development, as well as immune modulation of glucose and lipid metabolism and other biological processes, and is closely associated with diseases such as tumors, diabetes, and chronic inflammatory organ fibrosis (61). Previous studies have shown that miR-194-5p is closely related to residual β-cell function in children with type 1 diabetes mellitus (62). Recent studies have also found that miR-194-5p may participate in the progression of diabetic nephropathy by targeting ITGA9 to regulate macrophage migration and adhesion, thereby blocking the high glucose–induced upregulation of ITGA9 protein levels (63). Other studies have shown that the expression of *TGFB1*, *COL1A1*, and miR-139-5p changes in GDM patients, suggesting that miR-129-5p and miR-139-5p may play an important roles in GDM by regulating TGFB1 and COL1A1 gene networks (64). KCNQ1OT1 also plays an important role in regulating β-cell proliferation, scorching and insulin secretion, and cell death, as shown by Chen YL et al. (65). Studies have found that KCNQ1OT1 influences β-cell function by promoting its proliferation and insulin secretion, suggesting that it may serve as a new biomarker of islet function. However, in studies of type 2 diabetes caused by hepatitis C virus infection, it is shown that KCNQ1OT1 was found to promote the scorch death of β-cells infected by hepatitis C virus through the miR-223-3p/NLRP3 axis, thereby affecting insulin production and accelerating the onset of diabetes and (66). To date, there has been no study on the effect of KCNQ1OT1 on GDM, and its regulatory role of KCNQ1OT1 in GDM requires further investigation in the future.

In this study, three hub genes were used to predict related drugs. Four compounds—acetaminophen, nucleotide, dicrotophos, and lactic acid—were predicted by all three genes. Among these, dicrotophos is a highly toxic organophosphorus pesticide with teratogenic, embryotoxic, and neurotoxic properties. It is strictly prohibited for human or pregnancy-related research. Therefore, only the other three drugs will be discussed in the following section.

Previous studies have confirmed that prenatal use of acetaminophen is associated with adverse birth outcomes (67), but the correlation between acetaminophen and GDM still requires confirmation through animal experiments and clinical studies. Studies have shown that moderate administration of acetaminophen can activate the Nrf2 antioxidant pathway and reduce mitochondrial ROS generation (68). However, overdose induces hepatotoxicity. Targeted scavenging of mitochondrial ROS can significantly reduce drug-induced hepatotoxicity (69). These processes may correlate with the pathogenesis of GDM.

A recent study on the relationship between intestinal metabolic microflora and GDM in pregnant women showed that the changes in plasma lactate levels and hyperglycemia-related fecal microflora are associated with altered blood glucose levels in GDM patients, suggesting that modulation of intestinal microflora in pregnant women may help alleviate GDM (70). Lactic acid is an endogenous metabolite of glucose metabolism. When mitochondrial function declines, glycolysis is enhanced, leading to lactic acid accumulation. Lactic acid can activate the AMPK signaling pathway, thereby promoting mitochondrial biosynthesis (71–73). Therefore, lactic acid may participate in metabolic compensation by regulating hub genes, providing new insights into the mechanism of “glycolytic compensation for mitochondrial function” in the placenta of GDM. Studies have also shown that there are significant differences in the taxonomic composition of the oral microflora between GDM and non-GDM women. Metabolic pathway analysis revealed that 5-aminoimidazole ribonucleotide biosynthesis and inosine-5′-phosphate biosynthesis were enriched in the GDM women with GDM (74), suggesting that the oral nucleotide level in pregnant women may be closely related to the occurrence of GDM and could serve as a target for prevention and treatment of GDM. Nucleotides are the precursors for RNA synthesis. Mitochondria are prone to oxidative stress–related DNA damage, and nucleotide imbalance can lead to mitochondrial depletion due to reduced replication fidelity. Supplementation with nucleotides can promote the synthesis of mitochondrial ribosomal proteins by increasing the supply of mitochondrial transcription materials (75). Therefore, theoretically, ribonucleotide supplementation may improve the expression of mitochondrial ribosomal proteins through “material support,” potentially influencing the molecular mechanisms underlying GDM.

In this study, the hub genes related to mitochondria and immunity in GDM were identified using bioinformatics. By analyzing the relationship between the biological pathways of hub genes in bioinformatics and immune cells, we constructed the molecular regulatory network of these genes is constructed. However, there are still some limitations.

First, we used gene expression and co-expression network construction, but did not incorporate advanced data such as proteomics, which may limit a comprehensive understanding of the biological processes underlying GDM. To address this research gap, we plan to conduct detailed protein-level experiments in the future. Specifically, we will apply targeted proteomics techniques based on parallel reaction monitoring (PRM; high-sensitivity LC-MS/MS) and immunohistochemistry (IHC) or immunofluorescence (IF) to detect the protein abundance of MRPL15, MRPL22, and *MRPS18C*. Immunoprecipitation (Co-IP) will be used to verify key protein interactions and determine whether GDM disrupts mitochondrial ribosome assembly or its association with oxidative phosphorylation complexes.

At the same time, to correlate protein-level changes with actual mitochondrial function, we will use the Seahorse XF analyzer to assess mitochondrial respiratory parameters (such as basal respiration and maximal respiration), JC-1 staining to detect mitochondrial membrane potential, and the LC3B-II/LC3B-I ratio to evaluate mitochondrial autophagy levels.

Second, although the related hub genes were identified using machine learning algorithms and functional enrichment analyses, the dataset used in this study had a relatively small sample size, and the sample types between the training and validation sets were not consistent. Furthermore, no clinical validation was conducted on a large population or sample size. We recognize the necessity of more accurate and comprehensive clinical validation.

Therefore, further population-based experiments and clinical studies are essential. We plan to collaborate with three obstetrics and gynecology centers to collect samples from 150 women with GDM and 150 healthy pregnant women. Special attention will be given to paired sampling: collecting both primary placental artery cells and matched placental tissue samples from the same GDM/healthy participants. We will test whether the expression of MRPL15, MRPL22, and MRPS18C is consistent across sample types and extend the analysis to noncoding RNA and clinical levels by detecting the expression of related lncRNAs and miRNAs. Their association with clinical indicators—such as blood glucose and neonatal birth weight—will be analyzed to verify their potential as diagnostic biomarkers for GDM.

Finally, we currently lack experimental evidence directly linking hub gene expression changes to alterations in mitochondrial metabolic phenotypes. Therefore, we will conduct additional cellular-level experiments by constructing cell lines with gene knockdown or overexpression to verify the direct roles of these genes in regulating mitochondrial function and immune response, thereby further elucidating their mechanisms in GDM.

## 5 Conclusion

In this study, three hub genes related to mitochondrial and immune functions in GDM were identified using differential gene correlation and machine learning. The pathogenesis of GDM was explored through analyses of functional immune molecule regulatory networks and drug prediction, and further verified by animal models. These findings provide a foundation for the early diagnosis and treatment of GDM.

## Data Availability

The datasets presented in this study can be found in online repositories. The names of the repository/repositories and accession number(s) can be found in the article/supplementary material.
